# Supplementary material for: Mechanisms governing the pioneering and redistribution capabilities of the non-classical pioneer PU.1
Source: Nat Commun. 2020 Jan 21;11:402. doi: 10.1038/s41467-019-13960-2 (PMC6972792; doi:10.1038/s41467-019-13960-2)
Supplement: Supplementary file 7 — Source data [file 41467_2019_13960_MOESM7_ESM.zip › Source_Data/FigureS2/FigureS2B_MotifScanOutput/KmeansCluster13/homerResults/motif23.similar.html]

motif23

## Information for motif23

T
G
C
A
G
T
A
C
A
G
C
T
C
T
G
A
G
T
A
C
C
T
G
A
G
C
T
A
G
A
C
T
G
A
C
T
A
T
G
C
A
G
T
C
A
G
T
C
C
G
T
A
T
C
A
G
  
Reverse Opposite:  

A
G
T
C
A
C
G
T
A
C
T
G
A
C
T
G
A
T
C
G
C
T
G
A
C
T
G
A
C
G
A
T
A
G
C
T
A
C
T
G
G
A
C
T
C
T
G
A
A
C
T
G
A
C
G
T
  

|  |  |
| --- | --- |
| p-value: | 1e-23 |
| log p-value: | -5.349e+01 |
| Information Content per bp: | 1.789 |
| Number of Target Sequences with motif | 45.0 |
| Percentage of Target Sequences with motif | 2.66% |
| Number of Background Sequences with motif | 146.5 |
| Percentage of Background Sequences with motif | 0.32% |
| Average Position of motif in Targets | 89.5 +/- 50.8bp |
| Average Position of motif in Background | 90.7 +/- 69.6bp |
| Strand Bias (log2 ratio + to - strand density) | 0.6 |
| Multiplicity (# of sites on avg that occur together) | 1.13 |
| Motif File: | file (matrix) reverse opposite |

### Similar de novo motifs found

|  |  |  |  |  |  |  |  |
| --- | --- | --- | --- | --- | --- | --- | --- |
| Rank | Match Score | Redundant Motif | P-value | log P-value | % of Targets | % of Background | Motif file |
| 1 | 0.982 | T C G A G T A C A G C T C G T A G T A C T C G A G T C A G A C T A C G T A T G C A G T C A G T C C G T A | 1e-23 | -53.330955 | 2.72% | 0.35% | motif file (matrix) |
| 2 | 0.909 | T C G A T A G C A G C T T C G A G T A C G C T A G T C A G A C T C A G T A T G C A G T C T A G C | 1e-21 | -50.522945 | 3.78% | 0.78% | motif file (matrix) |
| 3 | 0.822 | T A G C A C G T C T G A T A G C C G T A T G C A A G C T C A G T A G T C G T A C A G T C | 1e-13 | -30.996377 | 1.95% | 0.34% | motif file (matrix) |
